# Informed Consent Practices for Publication of Patient Images in Dermatology Journals

**DOI:** 10.2196/60795

**Published:** 2025-04-18

**Authors:** Toluwani Taiwo, Bianca Obiakor, Sarah McClung, Kanade Shinkai

**Affiliations:** 1 School of Medicine University of California, San Francisco San Francisco, CA United States; 2 University of California, San Francisco San Francisco, CA United States; 3 Department of Dermatology University of California, San Francisco San Francisco, CA United States

**Keywords:** informed consent, patient images, patient consent, social media, dermatology, patient privacy, medical image

## Introduction

Clinical images play an important role in informing clinical care and education in dermatology. Standardized informed consent for publishing patient images is an important concern regarding patient privacy, especially given increasing avenues for dissemination (eg, online publication and social media) [1,2]. Protecting patient privacy is a critical aim for dermatologists, as publishing images with potentially identifiable features is often necessary. Establishing trust between dermatologists and patients is imperative when complete anonymity cannot be guaranteed [2]. Clear guidelines and thorough consent practices can ensure that authors are accountable for upholding patients’ privacy and are transparent when obtaining photo consent, thereby empowering patients to make informed decisions about sharing their images [3]. This study assesses current informed consent practices in image publication for top dermatology journals, examining author-facing guidelines and patient consent forms.

## Methods

In this cross-sectional study, we examined patient image submission guidelines and consent forms from the top 50 dermatology journals as defined by the 2023 Clarivate Journal Impact Factor ranking. We developed a checklist of image consent requirements informed by guidelines from the Declaration of Helsinki, International Committee of Medical Journal Editors (ICMJE), and Committee on Publication Ethics (COPE) as described in Multimedia Appendix 1 [4-6]. Between November 11 and 25, 2024, authors TT and BO reviewed journal websites to assess author requirements for image publication and examined patient consent forms when available. Checklist items were documented as present or absent in an Excel (Microsoft Corporation) spreadsheet. Criteria were considered met if explicitly stated in journal guidelines or consent forms, or if the Declaration of Helsinki, ICMJE, COPE, or publisher guidelines were explicitly referenced.

## Results

Among the 50 journals, 15 (30%) were published in the United States, 41 (82%) were indexed in MEDLINE (the National Library of Medicine’s primary bibliographic database and a component of PubMed), and 35 (70%) had a social media account on Facebook, X (formerly Twitter), Instagram, or LinkedIn. The median percentage of articles available through gold open access journal was 26% (IQR 14.1-78.8%). Results for image consent criteria from author-facing guidelines and patient consent forms are summarized in Tables 1 and 2.

**Table 1:** Image consent criteria listed in journal-specific author guidelines for the top 50 dermatology journals per the 2023 Clarivate Journal Impact Factor ranking.

Criteria for author-facing guidelines			Journals (N=50), n (%)
Requires informed consent to publish patient images			44 (88)
Specifies how image consent must be documented (eg, written statement on manuscript, letter of consent, or consent form)			41 (82)
Requires written consent from patient for publication of patient images			43 (86)
**Describes when image consent is necessary**			
	All patient images	24 (48)	
	Only images that are recognizable or contain identifying features	18 (36)	
**Statement about guidelines to which journal adheres**			
	Declaration of Helsinki	34 (68)	
	International Committee of Medical Journal Editors	29 (58)	
	Committee on Publication Ethics	36 (72)	
	Publishing group (Wiley, Elsevier, Taylor & Francis, and Springer)	25 (50)	
Specifies who can provide consent on behalf of patient (eg, parent/guardian if minor, next of kin)			33 (66)
**Provides guidelines for image modification**			40 (80)
	Eye bars or masking of eyes not permitted	34 (68)	
	Blurring of face/facial features not permitted	5 (10)	
	Cropping to exclude face/body parts permitted	5 (10)	
**Specifies identifiable features in patient images (eg, tattoos, birthmarks, jewelry, facial images)**			9 (18)
	Tattoos discussed	3 (6)	
	Birthmarks discussed	0 (0)	
	Jewelry discussed	1 (2)	
	Facial features/photos discussed	8 (16)	
Recommendations on authors’ storage of patient images			2 (4)
Statement about archiving/retaining patient publication consent			28 (56)
Patient review of manuscript required if identifiable features are present			13 (26)
Acknowledges possible dissemination of images on social media			3 (6)
**Has one or more social media handles**			35 (70)
	Facebook	23 (46)	
	X	32 (64)	
	LinkedIn	14 (28)	
	Instagram	15 (30)	
	Pinterest	0 (0)	
Journal- or publisher-specific consent forms provided			22 (44)

**Table 2:** Image consent criteria in consent forms of top 50 dermatology journals ranked by 2023 Clarivate Journal Impact Factor.

Criteria for journal/publisher image consent forms	Journals (n=22^a^), n (%)
Requirement to upload blank copy of consent form used if none is provided by the journal or publisher	4 (18)
Requirement to state consenting party and relationship to patient if consent is provided by proxy	19 (86)
Statement explaining why patient could not provide consent or lacked capacity if consent is provided by proxy	5 (23)
Form asks who explained and administered consent form to patient or proxy	21 (95)
Statement that signing the form does not waive patient’s right to privacy	4 (18)
Statement about the possibility of consent revocation^b^	10 (45)
Explicit mention of how images may be disseminated beyond print publication (eg, social media, internet)	17 (77)
Statement that journal cannot guarantee anonymity	13 (59)
Patient must provide written agreement to publication	20 (91)
Statement about the possibility of financial benefit	7 (32)
Form availability in multiple languages	2 (9)

^a^Only 22 of the top 50 dermatology journals provided consent forms per the 2023 Clarivate Journal Impact Factor ranking.

^b^Of the 22 journals with consent forms, 10 contained an explicit statement that consent may be revoked before the publication of a patient image, but not after.

## Discussion

This study highlights the lack of standardized patient image consent guidelines within dermatology journals. While most journals surveyed (n=44, 88%) required informed consent for patient image publication, only 44% (n=22) provided consent forms online, which could lead to heterogeneity in the process or documentation of obtaining consent. Among journals that offered a consent form, the inclusion of other key COPE guidelines varied. Taken together, differences in journal requirements regarding image modification, safeguards for protecting anonymity, and definitions of identifiable features could lead to ambiguity or variability in how institutions, researchers, and clinicians request informed consent which, in turn, could raise privacy concerns for patients [2,3].

Consent revocation policies were highly variable and were only explicitly stated in 45% (n=10) of journals. Importantly, some journals allowed revocation of consent only before publication. Additionally, a significant gap was seen in the few journals (n=3, 6%) with requirements regarding the disclosure of potential social media dissemination of published images, despite 70% (n=35) of journals having a social media presence on one or more major platforms.

This study was limited to a select number of dermatology journals, and potential interobserver variability was possible in the interpretation of published author guidelines. Additionally, whether journals enforce their stated privacy and consent requirements was not evaluated.

In conclusion, this study highlights a current lack of standardized requirements for publishing patient images in dermatology journals. This gap threatens patient privacy due to the potential for secondary uses and widespread online dissemination of published images, including via social media. These results identify important opportunities for journal editors to harmonize consent requirements among journals, including standardization of definitions of identifiable features, enhanced transparency about patient risks regarding the dissemination and secondary use of images online, and standards for obtaining patient consent.

## Appendix

Multimedia Appendix 1 List of the top 50 dermatology journals ranked by the 2023 Clarivate Journal Citation Report and a link to the publicly available raw dataset used in the study.

## Abbreviations

COPE: Committee on Publication Ethics
ICMJE: International Committee of Medical Journal Editors
