# Correction: Informed Consent Practices for Publication of Patient Images in Dermatology Journals

**DOI:** 10.2196/94194

**Published:** 2026-03-13

**Authors:** Toluwani Taiwo, Bianca Obiakor, Sarah McClung, Kanade Shinkai

**Affiliations:** 1 School of Medicine University of California, San Francisco San Francisco, CA United States; 2 University of California, San Francisco San Francisco, CA United States; 3 Department of Dermatology University of California, San Francisco San Francisco, CA United States

In “Informed Consent Practices for Publication of Patient Images in Dermatology Journals” [1], the authors made one correction.

In Multimedia Appendix 1, we included a Dryad link to our raw dataset, which includes Clarivate data. We have recently learned that Clarivate’s terms of use and Dryad’s data reuse policy are incompatible, and we will not be able to deposit the raw data in Dryad. The raw dataset can be made available by contacting the corresponding author.

In Multimedia Appendix 1, the following text has been revised:

The raw dataset for this study is available for review via the Dryad platform and accessible via the following unique link:http://datadryad.org/share/ k6Eiw-OfOGf0vJrxD6sOS8GC59H5SFuhtudjzHR1EH8.

The text now reads:

The raw dataset for this study is available by contacting the corresponding author.

The correction will appear in the online version of the paper on the JMIR Publications website, together with the publication of this correction notice. Because this was made after submission to PubMed, PubMed Central, and other full-text repositories, the corrected article has also been resubmitted to those repositories.

## Appendix

Multimedia Appendix 1 List of the top 50 dermatology journals ranked by the 2023 Clarivate Journal Citation Report and a link to the publicly available raw dataset used in the study.
