# Supplementary material for: Correction: Informed Consent Practices for Publication of Patient Images in Dermatology Journals
Source: JMIR Dermatol. 2026 Mar 13;9:e94194. doi: 10.2196/94194 (PMC13032088; doi:10.2196/94194)
Supplement: Multimedia Appendix 1 [file derma_v9i1e94194_app1.docx]

**Multimedia appendix for Informed Consent Practices for Publication of Patient Images in Dermatology Journals**

A checklist of journal image requirements was developed based on published recommendations from the Declaration of Helsinki (DH), International Committee of Medical Journal Editors (ICMJE), and Committee on Publication Ethics (COPE) [4-6]. The raw dataset for this study is available by contacting the corresponding author. Journals were confirmed as being indexed in MEDLINE through the National Library of Medicine (NLM) website (<https://www.ncbi.nlm.nih.gov/nlmcatalog>, accessed November 11, 2024). A Google search was performed to determine journals that had an identifiable handle and/or social media page on X (formally named Twitter), Facebook, Instagram, LinkedIn, and/or Pinterest. The average number of articles available as gold open access was obtained using the individual journal-specific average percentages provided by the 2023 Clarivate Journal Citation Report (<https://jcr.clarivate.com/jcr/home>, accessed November 11, 2024). The same website was used to obtain the list of the top 50 dermatology journals per the 2023 Clarivate ranking. The list of the top 50 dermatology journals used in the analysis is shown below.

**Top 50 Ranked Dermatology Journals per the 2023 Clarivate Journal Citation Report**

1. Journal of the American Academy of Dermatology
2. JAMA Dermatology
3. British Journal of Dermatology
4. American Journal of Clinical Dermatology
5. Journal of the European Academy of Dermatology and Venereology
6. Burns & Trauma
7. Journal of Investigative Dermatology
8. Advances in Wound Care
9. Journal der Deutschen Dermatologischen Gesellschaft
10. Psoriasis-Targets and Therapy
11. Contact Dermatitis
12. Mycoses
13. Dermatitis
14. Pigment Cell & Melanoma Research
15. Journal of Dermatological Science
16. Wound Repair and Regeneration
17. Actas Dermo-Sifiliográficas
18. Dermatologic Therapy
19. Clinical and Experimental Dermatology
20. Experimental Dermatology
21. Dermatology and Therapy
22. Acta Dermato-Venereologica
23. International Journal of Dermatology
24. Cosmetics
25. Burns
26. Indian Journal of Dermatology, Venereology & Leprology
27. Annales de Dermatologie et de Vénérologie
28. Journal of Cutaneous Medicine and Surgery
29. Dermatology
30. Journal of Dermatological Treatment
31. Journal of Dermatology
32. Skin Pharmacology and Physiology
33. International Journal of Cosmetic Science
34. International Wound Journal
35. Anais Brasileiros de Dermatologia
36. Dermatologic Surgery
37. Photodermatology, Photoimmunology & Photomedicine
38. Dermatology Practical & Conceptual
39. Journal of Tissue Viability
40. Current Dermatology Reports
41. Clinics in Dermatology
42. Journal of Cosmetic Dermatology
43. Dermatologica Sinica
44. Dermatology Reports
45. Lasers in Surgery and Medicine
46. Dermatologic Clinics
47. Australasian Journal of Dermatology
48. Cutis
49. Skin Research and Technology
50. European Journal of Dermatology
